# RNA Interference-Based Silencing of the *Chitin Synthase 1* Gene for Reproductive and Developmental Disruptions in *Panonychus citri*

**DOI:** 10.3390/insects11110786

**Published:** 2020-11-11

**Authors:** Muhammad Waqar Ali, Muhammad Musa Khan, Fang Song, Liming Wu, Ligang He, Zhijing Wang, Zhen-yu Zhang, Hongyu Zhang, Yingchun Jiang

**Affiliations:** 1 Institute of Fruit and Tea, Hubei Academy of Agricultural Sciences, Wuhan 430064, China; waqar3811@gmail.com (M.W.A.); fsong_ray@163.com (F.S.); wuliming2005@126.com (L.W.); lghe44@aliyun.com (L.H.); wzjjsz@sohu.com (Z.W.); 2 Key Laboratory of Bio-Pesticide Innovation and Application, Guangdong Province, South China Agricultural University, Guangzhou 510642, China; drmusakhan@outlook.com; 3 Key Laboratory of Horticultural Plant Biology (MOE), State Key Laboratory of Agricultural Microbiology, Institute of Urban and Horticultural Entomology, College of Plant Science and Technology, Huazhong Agricultural University, Wuhan 430070, China; zhangzhenyu@mail.hzau.edu.cn

**Keywords:** citrus red mites, sterility, parental RNAi, infertility, egg-hatch viability, dsRNA, RNAi, chitin regulation

## Abstract

**Simple Summary:**

The *Chitin Synthase 1* gene, when suppressed with RNAi, imparts differences in the structural development, physiology, and synthesis of epidermal chitin, which ultimately leads to the mortality of the target pest. The results presented here will help to illuminate the molecular mechanism and function of the *PcCHS1* gene, which regulates the egg-laying potential in adult citrus red mites. Using the leaf dip method, we found that dsRNA was potent and effective, and significantly reduced the egg-laying potential and hatching of citrus red mite eggs. These results show the potential utility of the *PcCHS1* gene in the development of novel RNA interference strategies for controlling the citrus red mite.

**Abstract:**

*Chitin synthase 1* (*CHS1*) is an essential gene regulating chitin during different developmental stages of arthropods. In the current study, we explored for the first time the role of *CHS1* gene regulation in the citrus red mite, *Panonychus citri* (McGregor) (Acari: Tetranychidae), by silencing its expression using (RNA interference) RNAi-based strategies. The results reveal that *P. citri* tested in different developmental stages, including larvae, protonymphs, deutonymphs, and adults fed on sweet orange leaves dipped in various concentrations (200, 400, 600, and 800 ng/μL) of dsRNA-*PcCHS1*, resulted in a continuous reduction in their gene expression, and the extent of transcript knockdown was positively correlated with the concentration of dsRNA. Concentration–mortality response assays revealed a mortality of more than 50% among all the studied developmental stages, except for adulthood. Furthermore, the target gene dsRNA-*PcCHS1* treatment of larvae, protonymphs, deutonymphs, and females at a treatment rate of 800 ng/mL of dsRNA significantly decreased the egg-laying rates by 48.50%, 43.79%, 54%, and 39%, respectively, and the hatching rates were also considerably reduced by 64.70%, 70%, 64%, and 52.90%, respectively. Moreover, using the leaf dip method, we found that the RNA interference effectively reduced the *PcCHS1* transcript levels by 42.50% and 42.06% in the eggs and adults, respectively. The results of this study demonstrate that the RNAi of *PcCHS1* can dramatically reduce the survival and fecundity of *P. citri*, but the dsRNA concentrations and developmental stages can significantly influence the RNAi effects. These findings indicate the potential utility of the *PcCHS1* gene in causing developmental irregularities, which could aid in the development of effective and novel RNAi-based strategies for controlling *P. citri*.

## 1. Introduction

The citrus red mite, *Panonychus citri*, is considered a major citrus pest [1,2]. It has a short life cycle and high reproduction capacity and can damage about 80 plant species, especially fruits and ornamental trees [3]. In recent decades, different strategies have been applied to control these pest populations, and acaricides were found to be the most efficient way to control the infestations of *P. citri*. Due to the unique adaptive characteristics of this pests and the overuse of acaricides, these pests have developed resistance against many acaricides [4]. Currently, the *P. citri* is one of the most dangerous citrus pests. It was shown to have developed a resistance of more than 17,000-fold in Japan against hexythiazox [5], and 601.5-fold in China against pyridaben [6]. Therefore, it is necessary to develop more efficient mite control measures.

Chitin is known as the second most abundant natural polysaccharide after cellulose. Chitin is abundant and is commonly found in nematodes, arthropods, and fungi [7,8]. The outer surfaces (exoskeletons) of arthropods are primarily composed of cuticles, which provide protection against environmental stress and facilitate movement [9]. However, this solid outer surface also imposes restrictions on the development and growth of pests. To avoid this situation, arthropods occasionally generate new cuticles to replace the old ones, a process in which chitin plays a key role [9]. Chitin-related genes have been reported in the ovaries, eggs, and egg shells of several arthropods [10,11,12].

The activity of chitin synthase (CHS) plays a vital role in regulating the chitin biosynthetic pathway in arthropods. The cDNA of the *CHS* gene has been widely cloned and characterized for different insect pest species [13,14,15]. However, relatively few researchers have focused on the genes regulating the chitin biosynthetic pathway in mites, especially *P. citri*. Generally, most pests harbor two types of chitin synthase genes, *CHS* class *A* and *CHS* class *B*, which correspond to *CHS1* and *CHS2*, respectively [16,17]. *CHS1* is considered to produce chitin that is used in cuticles and cuticular linings of the hindgut, foregut, and trachea during the developmental period. However, *CHS2* is produced explicitly by midgut epithelial cells and is associated with the peritrophic matrix [18,19]. CHS is a conserved enzyme which belongs to a family of two abundant glycotransferases found in organisms that synthesize chitin and are responsible for completing the biosynthetic chitin pathway.

RNA interference (RNAi) is a reliable gene silencing tool for studying gene functions by delivering gene-specific double-stranded RNA (dsRNA). This method is environmentally safe and known as a next-generation strategy for controlling pest populations [20,21]. In arthropods, successful delivery of dsRNA resulted in the silencing of the target gene, which ultimately led to the control or death of the target pests [22]. The successful arthropod pest control research findings allow us to assess the feasibility of using parental RNAi for the first time to control *P. citri*.

The current study was designed in consideration of the scarcity of knowledge on chitin-related genes in *P. citri* and their important roles in development. The study involved multiple experiments that principally aimed to (1) quantify the expression patterns of the target gene, *P. citri chitin synthase* (*PcCHS1*), in different developmental stages (eggs, larvae, protonymphs, deutronymphs, male adults, and female adults) of *P. citri*; (2) explore the silencing impact of the target gene dsRNA on the target gene; (3) evaluate the concentration-dependent responses of dsRNAs over the time in different developmental stages of *P. citri*; (4) determine the reproduction potential of *P. citri* under silenced *chitin synthase 1* gene conditions; (5) analyze the reduction in the chitin contents of exposed *P. citri* with the dsRNA of *chitin synthase 1*; and (6) observe the histological alterations in *P. citri* induced by the silenced *chitin synthase 1* gene in order to provide a chitin-regulation-based, novel option for the control of *P. citri*.

## 2. Materials and Methods

### 2.1. Rearing of Panonychus citri

Populations of *Panonychus citri* and fresh leaves were collected from an orchard of sweet oranges from a locality at the Huazhong Agricultural University (Hubei, Wuhan, China), that had not been sprayed with acaricide during the last ten years. The rearing protocol described in our previous study was followed by Joga [23]. Briefly, a susceptible strain (SS) of mites was kept under controlled conditions (28 °C ± 1 °C, 75–85% ± 5% RH and 12:12-h L:D) in a growth chamber. Fresh leaves (6 mm diameter) were put on a 7 mm layer of soft sponge, which was previously dipped in distilled water. *P. citri* were reared on these pieces of leaves [22]. The edges of the leaves were covered with dampened cotton to avoid the escape of *P. citri*.

### 2.2. Expression Profiles of the Target Gene

Prior to synthesizing the target gene dsRNA for suppression, it is important to make sure the target gene is expressed in all life stages of *P. citri*. Keeping in mind the importance of its expression in different life stages, we quantified the expression patterns of the *PcCHS1* gene in eggs, larvae, protonymphs, deutonymphs, and adults (males and females separately) with specific primers designed using the NCBI Primer-BLAST tool of the target gene, *PcCHS1* (*PcCHS1*-F-AACCGATATCTTCCCCGTTAT; *CHS1*-R-ACCTACATTCTTACTCTAGAT), and with *GADPH* as the internal control gene due to its stability in our preliminary study in different developmental stages of *P. citri* (*GADPH*-F-CAACCAATTGTCTTGCTCCTTTG, *GADPH*-R-CGGTAGCGGCAGGTATAATG) using an SYBR Green Master Mix in a Bio-Rad iCycler (Bio Rad, Hercules, CA, USA). In brief, total RNA to prepare three biological replicates was isolated from the pooled sample of each developmental stage (eggs, larvae, protonymphs, deutronymphs, males and females) of *P. citri* by TRIzol reagent (Invitrogen, Thermo Fisher, Waltham, MA, USA) [24]. The abovementioned sequence-specific primers of target genes were used to quantify their expressions under Bio-Rad iCycler, using Universal SYBR Green iTaq™ Supermix. In order to ensure that data met the statistical assumptions, the appropriate test was applied to check the normality before performing ANOVA. One-way ANOVA was used to analyze the expression patterns in different developmental stages of *P. citri* using GraphPad Prism 5.0. Significant differences among means were compared by Tukey’s HSD test (α < 0.05).

### 2.3. Preparation of Target Gene Double-Stranded RNA (dsRNA)

The dsRNA for RNAi was prepared by selecting a 318-bp target gene fragment from the gene *PcCHS1* (accession number KF241748), using T7+ *CHS1*-F-GGATCCTAATACGACTCACTATAGGGATACGGCGGCAAGTAACATC and T7+ *CHS1*-R-GGATCCTAATACGACTCACTATAGGAAGTTTGAATACGCGGTTGG primers. The control *egfp* fragment was amplified using *egfp* (EGFP_F+T7-GGATCCTAATACGACTCACTATAGGACGTAAACGGCCACAAGTTC; EGFP_R+T7-GGATCCTAATACGACTCACTATAGGAAGTCGTGCTGCTTCATGTG) primers from the Pub nls-*egfp* vector [25]. The target gene dsRNA was synthesized using a T7 RiboMAX™ Express RNAi System (Promega, Madison, WI, USA), according to the manufacturer’s protocol. After preparation, the dsRNA product was purified using MEGAclear™ (Ambion, Thermo Fisher) and stored at −80 °C for subsequent experimentation.

### 2.4. Sample Preparation for RNAi Bioassays

RNAi-mediated silencing of the target gene was conducted and evaluated among different life stages (eggs, larvae, protonymphs, deutonymphs, and adults) of *P. citri* following the previously published methodology [22]. Fresh leaves were washed in double-distilled water and subsequently cut into small pieces of equal size. After washing, the leaves were dried at a constant temperature of 55 °C for 5 min. The dsRNA-*PcCHS1* solutions of the target gene at different concentrations (200, 400, 600, and 800 ng/µL) were prepared using sterile distilled water, and leaves were dipped separately in the solutions for 1 h. The dsRNA-*PcCHS1*-dipped leaves were dried for 3 min in a laminar air flow cabinet. Then, the target gene dsRNA-*PcCHS1*-soaked leaves were immediately placed on water-saturated sponges, and 50 mites from each tested stage (eggs, larvae, protonymphs, deutonymphs, males, and females) per leaf were separately released. The control treatment was prepared using the control gene dsRNA-*egfp*-soaked leaves. Each experimental unit was incubated under the abovementioned controlled conditions. Three independent replicates were prepared in the same way.

### 2.5. Impacst of the Target Gene dsRNA on the Expression Patterns of Chitin Synthase 1 Gene Regulation in Different Stages of Panonychus citri

The total RNA from fifty *P. citri* in each developmental stage of the treated mites was isolated separately 24 h post-exposure using a TRIzol reagent [26], in order to prepare one replicate. Likewise, three biological replicates were prepared. In brief, 200 µL of TRIzol reagent was added into the 2 mL Eppendorf tubes containing 50 mites along with 2 beads of stainless. Tissue homogenizer was run for 30 s. After sample homogenization, 400 µL more TRIzol reagent was added and the Eppendorf tubes were kept on benchtop for 5 min for subsequent centrifugation at 12,000× *g*. After adding 100 µL chloroform, we kept the tubes on the benchtop for 2–3 min after mixing the chloroform. After centrifugation, the aqueous phase was washed using washing buffers in membrane spin columns. The purified total RNA was eluted by centrifugation at 10,000 rpm. A NanoVue spectrophotometer was used to determine the total RNA concentration, whereas the integrity of the RNA was checked using 2% agarose gel electrophoresis. The single-stranded cDNA was synthesized by a commercially available kit (Cat # K1612, Thermo Fisher) using 5 µg of total RNA. The qPCR analysis was performed using the Universal SYBR Green iTaq™ Supermix (Bio Rad) on a Bio-Rad iCycler, according to the manufacturer’s instructions. A 25µL reaction volume was used for the qPCR, containing 0.8 µL of each primer, 2 µL of cDNA, 6.4 µL of ddH_2_O, and 15 µL of the SYBR Green Master Mix. The thermal cycler amplification conditions were maintained as described by Ali et al. [22]. The qPCR data were analyzed according to Livak and Schmittgen [27]. In order to ensure that data met the statistical assumptions, the appropriate test was applied to check the normality before performing ANOVA. Stage-specific relative fold expression patterns of *P. citri* were analyzed by one-way ANOVA (Fisher’s LSD test, α < 0.05) using Statistix 8.1.

### 2.6. Time–Mortality Response of the Panonychus citri to Target Gene dsRNAs

The impact of the target gene dsRNA under laboratory conditions was evaluated by analyzing the time–mortality response of *P. citri* after being exposed to various concentrations (200, 400, 600, and 800 ng/µL) of dsRNA-*PcCHS1*, as mentioned in detail in the sample preparation section. In short, fifty each of larvae, protonymphs, deutonymphs, and adult mites were released on clean leaves separately after treatment. In case of control, mites were fed on leaves soaked separately in various concentrations of dsRNA-*egfp*, and three independent replicates were prepared in the same way. Each experimental unit was incubated at 28 °C ± 1 °C, 75–85% ± 5% RH, and 12:12-h L:D. After every 24 h, the mortality data were recorded until 9 d post-treatment. Mites were considered dead if no signs of movement were observed after touching with a fine brush. In order to ensure that data met the statistical assumptions, the appropriate test was applied to check the normality before performing ANOVA. A separate repeated-measures ANOVA with a Fisher’s post hoc test was performed to compare mortality among dsRNA concentrations and time points for each developmental stage.

### 2.7. Effects of Target Gene dsRNA on the Reproduction Potential of the Panonychus citri

The impacts of the target gene suppression on the fecundity and subsequent egg-hatch viability were evaluated by exposing fifty larvae, protonymphs, deutonymphs, and newly matured adult female mites separately on each leaf treated with dsRNA-*PcCHS1* at a concentration of 800 ng/μL. In the case of the control treatment, dsRNA-*egfp* was used as the treatment solution. Three replicates were prepared likewise. Mites were shifted to new fresh leaves after 24 h, until they reached the adult stage, in order to observe the life stage-specific and prolonged impact of the target gene dsRNA-*PcCHS1* on the reproduction potential of citrus red mites. After 24 h of feeding on the dsRNA-treated leaves, depending upon the stage of mites, the newly matured exposed females were allowed to mate with untreated males after they reached adulthood. The mites were shifted to new fresh leaves after 24 h, whereas old leaves were kept in an incubator to count the number of eggs in order to calculate their egg-hatch viability. The hatching rate was assessed after eight days. However, the leaves were changed every 24 h for eight days. In order to ensure that data met the statistical assumptions, the appropriate test was applied to check the normality before performing ANOVA. The egg-laying and egg-hatch viability data were analyzed separately for each treated stage using a repeated measures ANOVA, and significant differences among the means were analyzed by a Fisher’s LSD test.

### 2.8. Impact of the Target Gene dsRNA on the Chitin Contents of Panonychus citri

The chitin contents of *P. citri* eggs and adults in response to 800 ng/μL of dsRNA-*PcCHS1* (treatment) and dsRNA-*egfp* (control) that were previous applied during late deutronymph stage were evaluated for chitin extraction, following the alkaline deacetylation method. Overall, three replicates were prepared, and each replicate was prepared by pooling 20 mg of mite samples. The chitin contents in the samples were determined at 650 nm in a spectrophotometer [28]. The data were analyzed using a one-way ANOVA, and significant differences among means of chitin contents in different developmental stages were analyzed by a Fisher’s LSD test.

### 2.9. Histological Analysis to Observe Alterations

The late deutronymph stage *P. citri* fed on leaves soaked with target gene and control dsRNA were used for histological analysis. In brief, after dewaxing the paraffin parts, mite sections were transferred through a series of solutions, including two washes with xylene solution for 20 min each wash, two washes with 100% ethanol for 5 min each wash, a 5 min wash with 75% ethanol, and a 5 min water wash. After washing, immersion was performed with hematoxylin and eosin staining using a hematoxyl solution, with multiple rinses and washes. After removing the slides from the xylene, they were dried for subsequent observation under a NIKON Eclipse Ti-SR microscope (Tokyo, Japan), and a digital camera (D3500 DSLR, Tokyo, Japan) was used to take images [29].

For transmission electron microscopy, protonymphs, deutronymphs, and adults fed on leaves treated with target gene and reference gene dsRNAs for about 36 h were prepared by following the standard methodology. The samples prepared for visualization were observed using a HT7700-SS Transmission electron microscope (HITACHI, Tokyo, Japan).

In order to label chitin, samples were labeled using Calcofluor, after the removal of paraffin. Calcofluor was thus used to visualize differences in the chitin material. The slides were washed three times with a PBS buffer, and the initial fluorescent Calcofluor solution (Sigma-Aldrich, St. Louis, MO, USA) was diluted to a final concentration of 10 μg/mL. The samples were incubated for 120 min. Once the parts were washed three times with PBS for half an hour each, the slides were observed under an Olympus BX51 fluorescent microscope (Tokyo, Japan).

## 3. Results

### 3.1. Expression Profile of the PcCHS1Gene in Different Developmental Stages of the Panonychus citri

**Figure 1:**
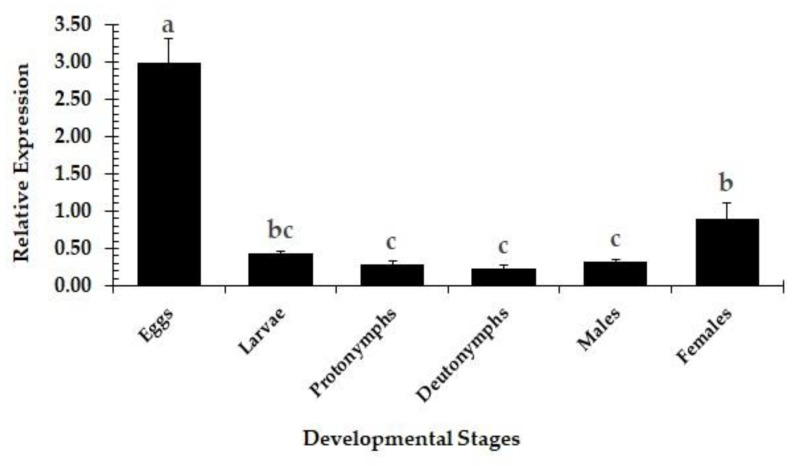
Relative expression patterns of the *P. citri Chitin Synthase 1* (*PcCHS1*) in different developmental stages. The treatment bars show the mean value of three independent replications (*n* = 50 per replicate), while the error bars show the mean deviation. Different lower-case letter(s) above the bars indicate significant differences in the relative expression of the target gene (Tukey’s HSD test, α < 0.05). Different small case letters on the bars showing significant difference between the treatments.

The qRT-PCR based analysis of gene expression in eggs, larvae, protonymphs, deutonymphs, male adults, and female adults (Figure 1) revealed that the transcripts of *PcCHS1* were expressed in all the tested developmental stages. The expression of *PcCHS1* was significantly higher in eggs, followed by female adults. The gene expression in the other four developmental stages (larvae, protonymphs, deutonymphs, and male adults) remained significantly lower (Figure 1).

### 3.2. RNAi-Based Silencing of PcCHS1 in Different Developmental Stages of the Panonychus citri

**Figure 2:**
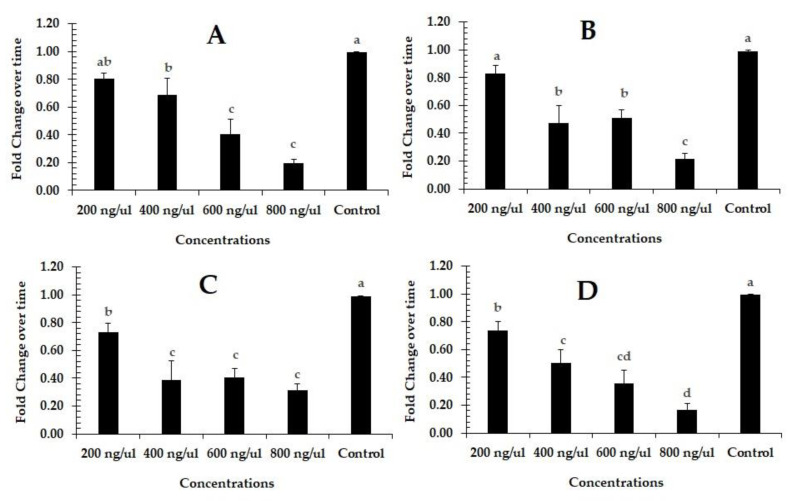
The effect of RNAi-mediated efficacy and silencing of target genes in different developmental stages of citrus red mites: (**A**) larvae; (**B**) protonymphs; (**C**) deutonymphs; and (**D**) adults at different concentrations (200, 400, 600, and 800 ng/µL) of dsRNA-*PcCHS1* using the leaf dip method. The normalized gene expression is shown in comparison to the dsRNA-*egfp* control (set to 1-fold). The treatment bars show the mean value of three independent replications (*n* = 50 per replicate), while error bars show the mean deviation. Different lower-case letter(s) above the bars indicate significant differences in the relative expression of the target gene (Fisher’s LSD test, α < 0.05). Different small case letters on the bars showing significant difference between the treatments.

The qRT-PCR results show that “pcCHS1-dsRNA” delivery using the leaf dip method at 600 and 800 ng/µL was the most effective, compared to the control group (dsRNA-*egfp*). After 24 h of exposure, the expression of the target gene in the larvae showed a level of 0.80, 0.68, 0.40, and 0.19 of *CHS1* transcript relative to the control in response to 200, 400, 600, and 800 ng/µL of ds-*PcCHS1*, respectively (*F* = 18.00; df = 4, 10; *p* = 0.0001). The maximum down-regulation (0.31-fold) of the *PcCHS1* gene was observed in adults after 24 h of exposure at a dsRNA concentration of 800 ng/µL, compared to the control (1-fold) (*F* = 22.90; df = 4, 10; *p* = 0.0001). The level of the *PcCHS1* gene decreased by 0.73-, 0.38-, 0.40-, and 0.31-fold relative to the control gene in deutonymphs (*F* = 14.10; df = 4, 10; *p* = 0.0004), and by 0.83-, 0.47-, 0.50-,and 0.21-fold relative to the control gene in protonymphs (*F* = 18.90; df = 4, 10; *p* = 0.0001), in response to 200, 400, 600, and 800 ng/µL of dsRNA, respectively (Figure 2A–D). The differential effect of the target gene (*PcCHS1*) dsRNA at concentrations of 400, 600 and 800 ng/µL was found to be at the same level of significance in deutronymphs (Figure 2C). 

### 3.3. Time–Mortality Response in Different Developmental Stages of the Panonychus citri

**Figure 3:**
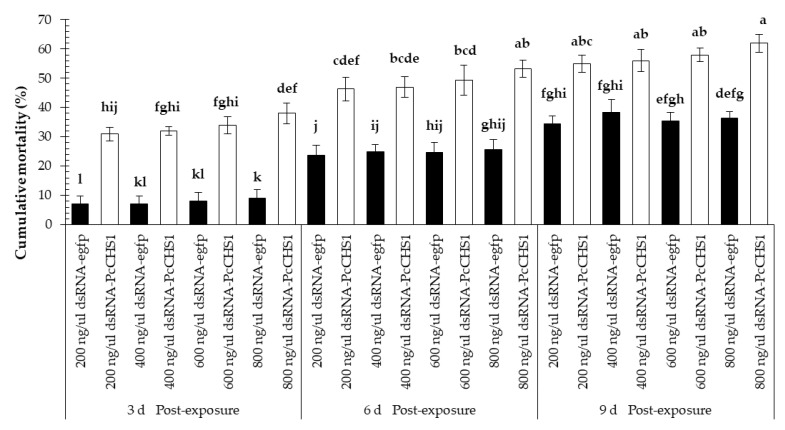
Time-mortality response of *P. citri* larvae upon exposure to the target gene (*PcCHS1*), dsRNA-*PcCHS1*, compared to the control group (dsRNA-*egfp*). The values are the means of three independent replicates (*n* = 50 per replicate). The mortality response over time was analyzed by a repeated measures ANOVA, and significant differences in the means were analyzed by a Fisher’s LSD test (α = 0.05). Different small case letters on the bars showing significant difference between the treatments.

The dsRNAs of the target gene caused considerable mortality among the larvae of *P. citri*. Significant differences in the larval mortality were observed at all the recorded time intervals (*F* = 17.65; df = 2, 42; *p* = 0.0031), treatments (*F* = 101.03; df = 7, 42; *p* = 0.00001), and interactions (*F* = 3.71; df = 14, 42; *p* = 0.0005). Overall, *P. citri* fed on leaves treated with dsRNA-*PcCHS1* showed a relatively higher mortality and remained at a significantly higher level compared with the control treatment, in which dsRNA-*egfp* was used to treat leaves fed to the control population (Figure 3). Furthermore, the mortality response among larvae was enhanced over the time and showed a directly proportional, concentration-dependent mortality relationship at most of the provided time intervals, as shown in Figure 3.

**Figure 4:**
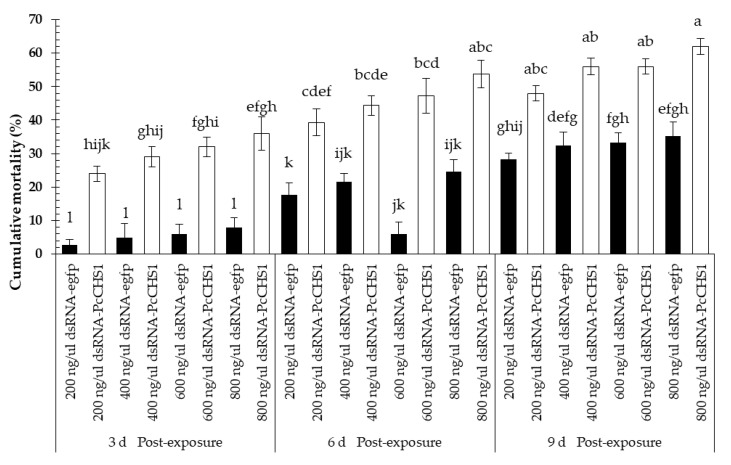
Time–mortality response of *P. citri* protonymphs upon exposure to the target gene (*PcCHS1*) dsRNA-*PcCHS1*, compared to the control group (dsRNA-*egfp*). The values are the means of three independent replicates (*n* = 50 per replicate). The mortality response over time was analyzed by a repeated measures ANOVA, and significant differences among the means were analyzed by a Fisher’s LSD test (α = 0.05). Different small case letters on the bars showing significant difference between the treatments.

Protonymphs of *P. citri* fed on leaves exposed to different concentrations of the control dsRNA-*egfp* and target gene dsRNA-*PcCHS1* revealed a noticeable time-dependent mortality response (Figure 4). However, we recorded significant differences in the mortality of *P. citri* recorded at various time intervals (*F* = 20.67; df = 2, 42; *p* = 0.0020), using various concentrations (*F* = 100.00; df = 7, 42; *p* = 0.00001) and interactions (*F* = 4.06; df = 14, 42; *p* = 0.0002). The highest concentration (800 ng/μL) of dsRNA-*PcCHS1* was able to cause a mortality >60% among protonymphs. Overall, the highest mortality was recorded for experimental mites fed on leaves exposed to dsRNA-*PcCHS1*.

**Figure 5:**
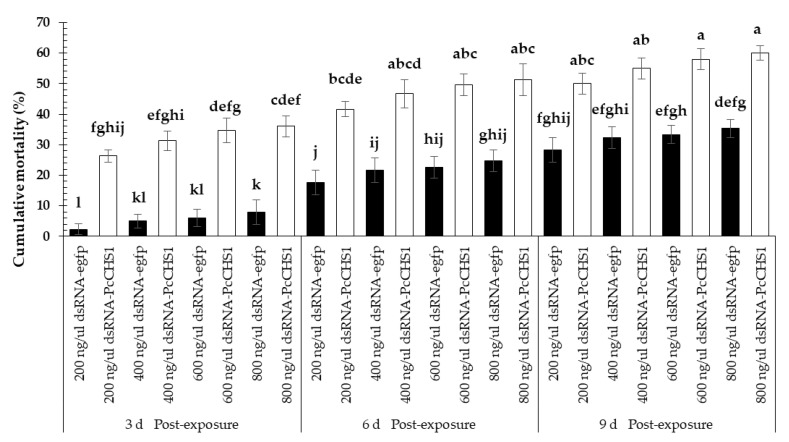
Time–mortality response of *P. citri*. deutonymphs upon exposure to the target gene (*PcCHS1*) dsRNA-*PcCHS1*, compared to the control group (dsRNA-*egfp*). The values are the means of three independent replicates (*n* = 50 per replicate). The mortality response over time was analyzed byarepeated measures ANOVA, and significant differences among the means were analyzed by a Fisher’s LSD test (α = 0.05). Different small case letters on the bars showing significant difference between the treatments.

Time-mortality response bioassays conducted on *P. citri* by exposure of deutonymphs on dsRNA revealed significant differences among their mortalities, which were recorded at different time intervals (*F* = 16.88; df = 2, 42; *p* = 0.0034) and using different concentrations of the target (dsRNA-*PcCHS1*) and control genes (dsRNA-*egfp*) (*F* = 130.07; df = 7, 42; *p* = 0.00001) and interactions (*F* = 5.28; df = 14, 42; *p* = 0.00001). During the first three post-exposure days, an eligible mortality was recorded in the control treatment. The results also show a trend towards time-dependent mortality response. However, the enhanced mortality response compared to the control was obvious during the first three days of exposure, as the highest concentration caused a mortality of >35%inthe deutonymphs of *P. citri* (Figure 5). Overall, the highest mortality (approximately 60%) was recorded in the treatment of deutonymphs of *P. citri* fed on the highest concentration of the target gene dsRNA-*PcCHS1* at the 9 d time point.

**Figure 6:**
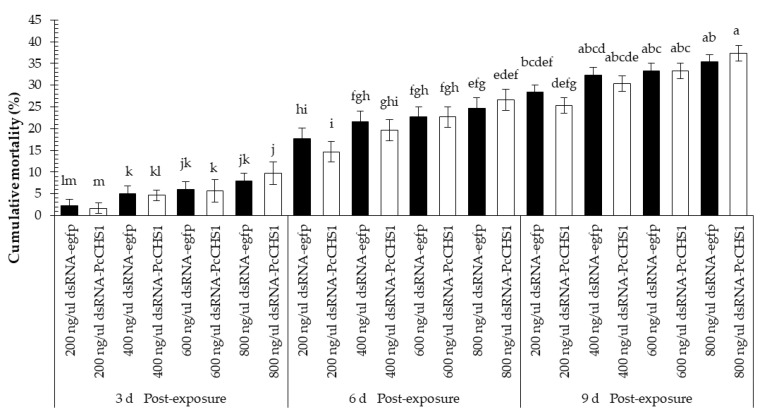
Time–mortality response of *P. citri* adults upon exposure to the target gene (*PcCHS1*) dsRNA-*PcCHS1*, compared to the control group (dsRNA-*egfp*). The values are the means of three independent replicates (*n* = 50 per replicate). The mortality response over time was analyzed by a repeated measures ANOVA, and significant differences among the means were analyzed by a Fisher’s LSD test (α = 0.05). Different small case letters on the bars showing significant difference between the treatments.

In this study, adults were found to be the least responsive to RNA-mediated suppression of *PcCHS1* and revealed the lowest mortality response among *P. citri* at this development stage. Even the highest concentration of dsRNA failed to cause 50% mortality (Figure 6). Overall, the adult mortality results reveal significant differences recorded after 3, 6, and 9 d post-exposure (*F* = 36.90; df = 2, 42; *p* = 0.0004) under various treatments (*F* = 81.45; df = 7, 42; *p* < 0.00001) and interactions (*F* = 2.83; df = 14, 42; *p* = 0.0046).

### 3.4. Impact of Target Gene Silencing on the Reproduction Potential of Panonychus citri

**Figure 7:**
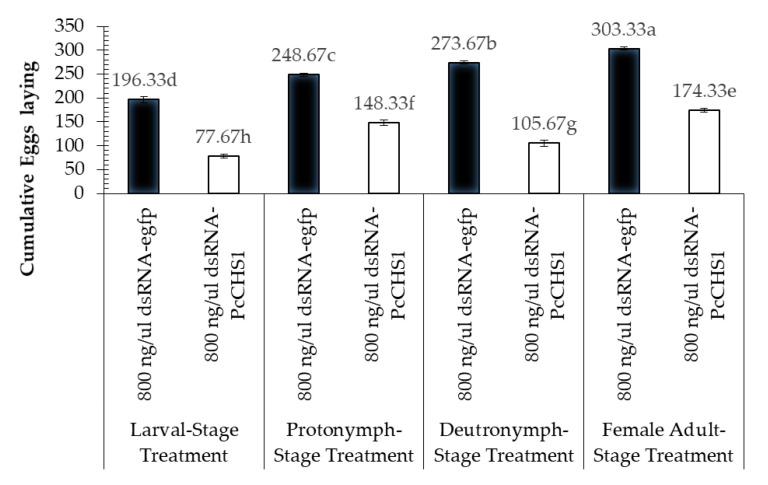
Fecundity of *P. citri* treated with the target gene dsRNA in different developmental stages. The values are the means of three independent replicates. The mortality response over time was analyzed by a repeated measures ANOVA, and significant differences among the means were analyzed by a Fisher’s LSD test (α = 0.05). Different small case letters on the bars showing significant difference between the treatments.

The down-regulation of the target gene revealed interesting patterns in the cumulative number of eggs laid by the females and their hatching. The RNAi of the target gene dsRNA *PcCHS1* showed a significant reduction in egg-laying at all life stages (larvae, protonymphs, deutronymphs, and adults) when fed leaves dipped in dsPcCHS1 compared to leaves dipped in dsRNA-*egfp* (Figure 7). Overall, all the treated developmental stages of *P. citri* exposed to the target gene *PcCHS1* remained significantly less abundant, compared to the *P. citri* of the control treatment (dsRNA-*egfp*) (Figure 7). However, significant differences in the cumulative egg laying were recorded in the different developmental stages of *P. citri* (*F* = 481.41; df = 3, 12; *p* = 0.00001), treated with different treatments (*F* = 456.44; df = 1, 12; *p* = 0.00001) and interactions (*F* = 56.03; df = 3, 12; *p* = 0.00001).

**Figure 8:**
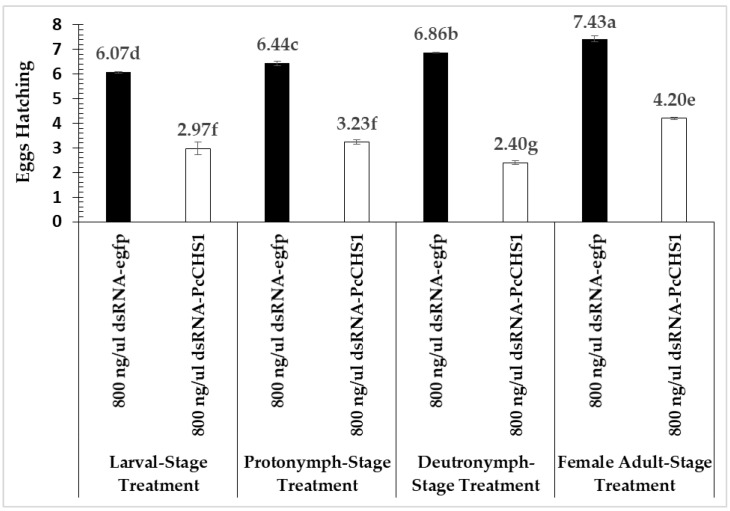
Cumulative egg hatching of *P. citri* treated with the target gene dsRNA during different developmental stages. Bars represent means and standard errors calculated from three independent replicates. The mortality response over time was analyzed by a repeated measures ANOVA, and significant differences among the means were analyzed by a Fisher’s LSD test (α = 0.05). Different small case letters on the bars showing significant difference between the treatments.

Significant differences in the egg-hatch viability of *P. citri* eggs were recorded from the females of *P. citri* exposed during different developmental stages (*F* = 50.64; df = 3, 12; *p* = 0.0002), treated with different treatments (*F* = 1903.54; df = 1, 12; *p* = 0.00001) and interactions (*F* = 15.01; df = 3, 12; *p* = 0.0002). The lowest egg hatching rate was recorded in the *P. citri* treated with 800 ng/μL of dsRNA-*PcCHS1* during their deutonymphal stage (Figure 8). However, the control treatment (dsRNA-*egfp*) remained at a statistically higher level, compared with the target gene treatment, during all the tested developmental stages.

### 3.5. Disruptions in Chitin Contents

**Figure 9:**
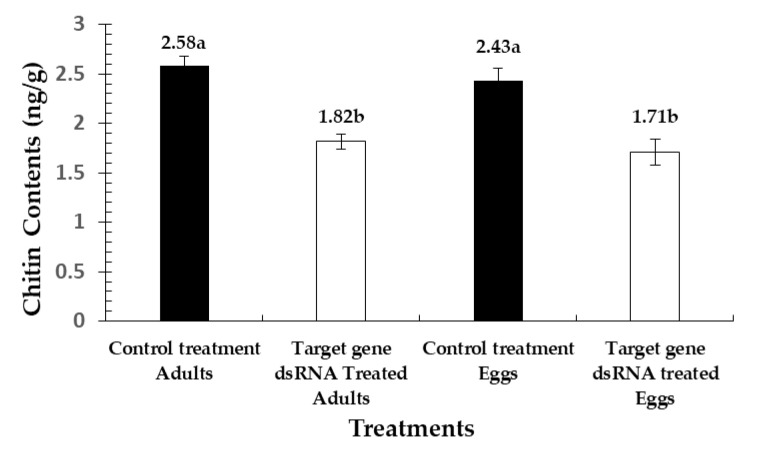
Chitin contents recorded in different developmental stages of *P. citri*. The values are the means of three biological replicates. Bars represent means and standard errors calculated from three independent replicates. Each replicate was compiled by pooling mites to make a 20 mg sample for analysis. The chitin contents were analyzed by ANOVA, and significant differences among the means were analyzed by a Fisher’s LSD test (α = 0.05). Different small case letters on the bars showing significant difference between the treatments.

The chitin contents of *P. citri* were determined in two developmental stages (eggs and adults), as shown in Figure 9. The results show a significant reduction (*F* = 16.10; df = 3, 11; *p* = 0.0009) among the exposed mites, compared to the control. Reductions of 42.50% and 42.06% were noted in the chitin contents of eggs and adults, respectively, compared to the control treatment (ds-RNA-*Pc-egfp*).

### 3.6. Cuticular Disruption and Cellular Abnormalities

**Figure 10:**
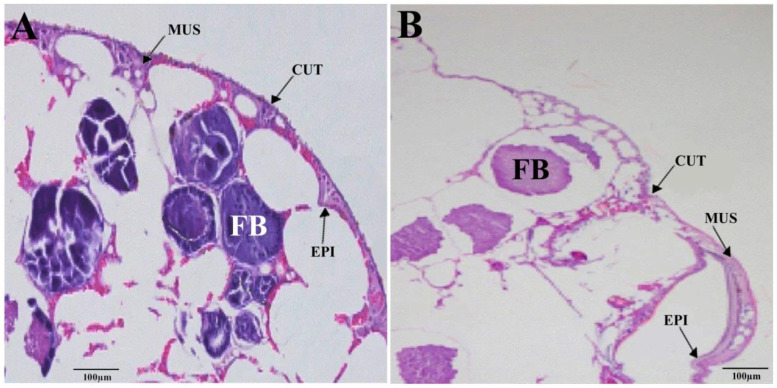
Hematoxylin and eosin staining of adult mites from the (**A**) (control gene dsRNA-*egfp*) and (**B**) (target gene dsRNA-*PcCHS1*) treatment groups. CUT stands for cuticle; EPI stands for epidermis; FB stands for fat bodies; and MUS stands for muscles. CUT and FB indicate cuticle and fat bodies, respectively.

Microscopic analysis revealed that the entire dsRNA-*PcCHS1*-treated adults integument was thinner and impaired, compared with the dsRNA-*egfp* control group (Figure 10). The cuticle layers of all the treated populations were broken, shrinking, transparent, apart from the epidermis, and deficient in cuticular cells (Figure 10).

### 3.7. Chitin Reduction in the Cuticles of Panonychus citri

**Figure 11:**
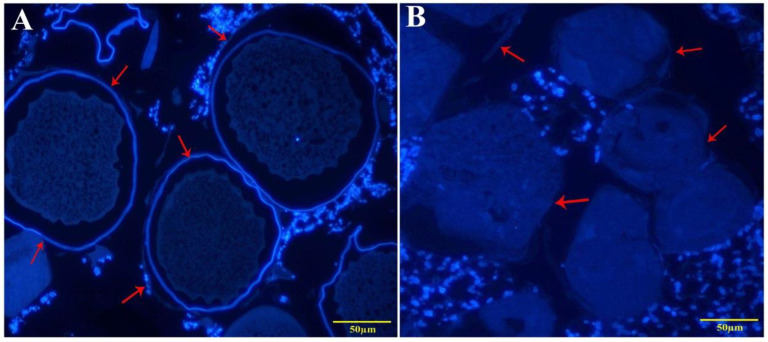
Fluorescence microscopy of the cuticular chitin presence in adult *P. citri* treated with (**A**) dsRNA-*egfp* (control gene); and (**B**) dsRNA-*PcCHS1* (target gene). The arrow heads indicate the alterations in the epidermises and cuticles of *P. citri* adults.

The microscopic analysis revealed a normal and continuous calcofluor labeling in the case of the integument of the control group of adults (Figure 11A). On the other hand, *P. citri* treated with the target gene dsRNA-*PcCHS1* were rough, irregular, and scattered, as seen in Figure 11B. The pigmentation of calco fluorine under fluorescence was low in the case of target gene silencing, which enabled us to suggest that target gene silencing considerably reduced the cuticular chitin contents (Figure 11).

### 3.8. Transmission Electron Microscopic Analysis

**Figure 12:**
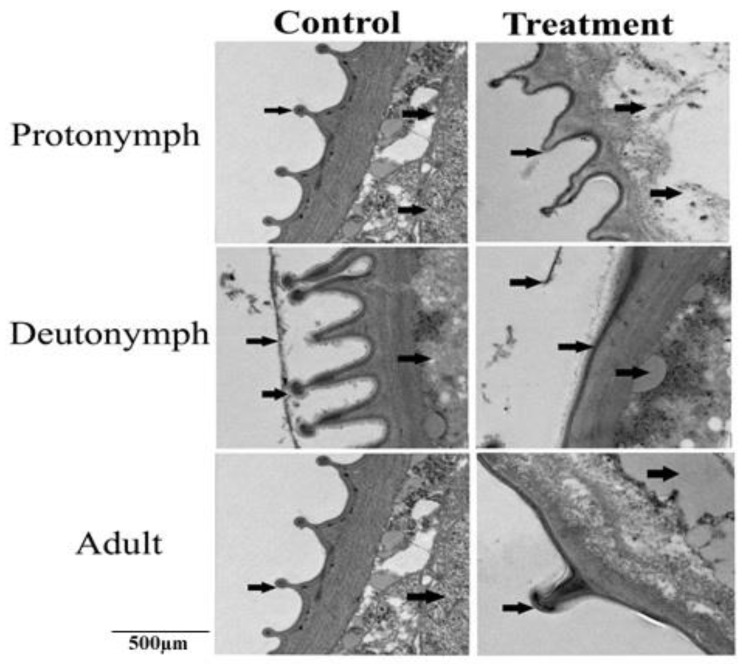
Transmission electron microscopic (TEM) analysis of the body wall denticles and tracheal taenidia at different developmental stages of *P. citri* treated with the target gene dsRN-*PcCHS1* and control gene dsRNA-*egfp*. Arrows indicate the disturbance of chitin and ecdysial droplets, exhibiting a separation from the apical plasma membrane. Scale bar 500 µm.

The role of the down-regulation of *PcCHS1* genes caused by their dsRNA-*PcCHS1* through RNAi in the cuticular chitin maintenance of *P. citri* during different developmental stages, including protonymphs, deutonymphs, and adults, was analyzed by transmission electron microscopy. There was a reduction in the chitin contents after dsRNA-*PcCHS1* treatment at the protonymph, deutonymph, and adult stages. These findings enable us to suggest that the *PcCHS1* gene is very important and plays a key role in the regulation of the chitin level in the cuticle, specifically at the adult stage of the development of *P. citri* (Figure 12). The images at the apical plasma membrane clearly revealed degraded enzymes due to the presence of ecdysial droplets, exhibiting a separation from the apical plasma membrane. This process goes on while the ecdysial space starts to emerge. The ecdysial droplets, prior to ecdysis, disappear once the new cuticle is synthesized, as can be seen in Figure 12.

## 4. Discussion

In the present study, a specific gene (*PcCHS1*) was silenced in *P. citri*, and the effectiveness was determined using the dsRNA leaf dip method. The experimental results reveal that the tested dsRNA of *PcCHS1* had significant effects on mite mortality and female infertility. Additionally, to examine the impact of the target gene dsRNA on different developmental stages, the most effective concentration was chosen for the production of infertile females. The highest concentration of the target gene (*PcCHS1*) dsRNA (800 ng/µL) resulted in a mortality of more than 50% in larvae, protonymphs, and deutonymphs. The parental RNAi strategic application of the target gene dsRNA caused 54%, 39%, and 43.9% reductions in egg laying in protonymphs, deutonymphs, and females, respectively. Interestingly, our findings also show a significant decrease (64%) in egg hatching, compared to the control, which could aid in the development of a novel gene-specific and environmentally friendly strategy of using parental RNAi against *P. citri*.

The expression of the target gene in different developmental stages was investigated. The target gene was highly expressed in eggs and females; however, very low expression was observed in protonymphs and deutonymphs. The high expression levels in our data are very similar to previously reported results [30] (Figure 1).

The effect of the *PcCHS1* target gene dsRNA was recorded in different developmental stages of *P. citri*, and significant mortality in larvae, protonymphs, and deutonymphs was observed, compared to the control group (dsRNA-*Pc-egfp*). Chitin synthesis inhibitors (e.g., diflubenzuron, methoprene) are chemicals that inhibit chitin synthesis, and ultimately cause adult mortality in different pest species [31]. Nymphs or larvae might not digest old cuticles and thus can produce mild and malformed cuticles, resulting in mass mortality [32]. As the chitin biosynthetic pathway is absent in vertebrates and humans, chitin inhibitors have high potential for integrated pest management (IPM) of *P. citri* in citrus fields. Previous results show that exposure to chitin inhibitors could reduce the chitin content due to the inhibition of CHSI activity or reduction in mRNA abundance, which may indicate the presence of a regulatory response mechanism that restores the enzyme content [32]. In our experiments, a high mortality of nymphal instars was observed, compared to the controls. This may be because *PcCHS1* is essential for the development of outer chitin and the maintenance of other metabolic functions. Our results are similar to those found in a previous study, where a chitin inhibitor caused a high nymphal mortality (100%) after one week of exposure to the chitin inhibitor [33]. On the other hand, adult mortality in the present study was found to be lower (<50%) compared with other developmental stages. This could be perhaps overcome with additional optimization including increasing dsRNA doses, longer exposure times, targeting of more than one gene in the chitin synthase pathway, formulation, etc. in order to enhance suppression in the *CHS1* gene for economic feasibility.

The target gene dsRNA significantly impacted on eggs lying compared to the control group. The results of the present study are in line with previous studies, which revealed that eggshell amalgamation occurs in two phases, and ds-*PcCHS1* strongly down-regulates chitin synthesis. All of these effects led to the disturbance of egg deposition [34]. A significantly reduced egg laying rate in response to the inhibition of *PcCHS1* has been previously reported in different pest species [35,36,37]. However, Arias et al. (1975) did not find any significant difference in the number of eggs laid by *Culex tarsalis* females treated with the chitin inhibitor diflubenzuron. A difference in the number of eggs laid by surviving *Aedes aegypti* females after methoprene treatment was also non-significant, compared with controls [38]. Our results reveal that larvae, protonymphs, deutronymphs, and adults fed on dsRNA-*egfp*-dipped leaves also showed significantly different cumulative egg laying and hatch rates. These findings are in agreement with previous studies showing reduced fecundity in *Nilaparvata lugens* Stål (Hemiptera: Delphacidae) compared with a normal population [39].

RNAi is a useful molecular biological technique for exploring gene function through mRNA knockdown [40]. In arthropods (insects), RNAi has been widely used to knock down chitinase genes [41]. RNAi triggered by dsRNA has proven its potential against sucking pests [23,42]. In the current study, dsRNA exposure via the leaf dip method effectively knocked down the *PcCHS1* gene, which led to a significant reduction in the chitin content in eggs and adults. Our results can also be justified by a previous study, where the mRNAs of two chitinase genes (*SeChi* and *SeChi-h*) were knocked down in *Spodoptera exigua*, causing phenotypic defects in the pupa and eclosion stages [43]. Similar results were also observed in *Tribolium castaneum* by injecting dsRNA [41]. The silencing of the *PcCHS1* gene led to a reduction in the chitin content in eggs and adults, resulting in mortality and reduced fecundity. However, during that study, no significant phenotype differences were observed, as in this study [41,44]. The current study also indicates that *chitin synthase 1* (*PcCHS1*) is crucial for survivorship, fecundity, and other physiological processes. Therefore, the study of other chitin pathway genes in *P. citri* may uncover the functions of these multiple genes in the future.

The dsRNA treatment substantially decreased the expression of *PcCHS1* and reduced the number of eggs laid, along side the histological and phenotypical changes in different developmental stages of *P. citri*. The arrow shows that the cuticle layer was missing (Figure 11). The ds-*PcCHS1*-treated mites displayed abnormal cell structure and disturbed circular layers compared to the control group, and a reduction in chitin was evident in this study, proving the function of *PcCHS1* in development and morphology. We conclude that the *PcCHS1* gene has an integral role not only in synthesizing chitin, but also in *P. citri* development.

Insect growth regulators (IGRs) are substances that interact in standard life cycles and insect development [45]. Chitin synthesis inhibitors (*CSIs*) are among the IGRs that affect normal growth and development, and prevent the formation of chitin [46]. Previous studies showed this by subjecting *A. aegypti* and *Bomix mori* to CSI. A physiological change and depletion of chitin were observed, with a lower *CHS1* expression in the integument [47,48]. The *CHS1* mutations also caused major cuticular disruption in *Drosophila* [49]. Likewise, CSIs inhibit oviposition and have significant reproductive consequences [49]. Diflubenzuron, for instance, alters growth and survival and inhibits *D. melanogaster* reproduction, while a sublethal dose of hexaflumuron and teflubenzuron substantially reduced the oviposition, fecundity, fertility, development, and disruption of *Helicoverpa zea*, *A. aegypti*, *Plutella xylostella*, and *Locusta migratoria* [46,50,51]. Chitin development is a complicated process, and *CHS1* is the main enzyme sequenced in many species of pests [52]. CSI blocks the proliferation of chitin subunits through ion transfer channels. CSI binds transporters from the sulfonylurea receptor (SUR) and ATP-binding cassette (ABC) transporter families, and blocks the chitin synthesis by inhibiting *CHS1* activity [53]. Chitin synthesis requires many factors, which serve as precursors of chitin synthesis in pests [49]. *Tre-1* (*trehalase 1*) and *Tre-2* (t*rehalase 2*) are found in tissues that contain chitin, such as the epidermis and trachea. SeTre-1 dsRNA in *S. exigua* decreased *CHS1* gene transcripts, resulting in a mortality of 50–60%, and it had a high impact on cuticular chitin [54]. The expression of the gene *Treh-1* in *O. fuscidentalis* increases after treatment with JH acid or 20E [55]. In addition, the *UAP1*(*UDP-N-Acetylglucosamine Pyrophosphorylase 1*) gene also plays a major role in cuticle chitin synthesis, and knockdown has resulted in the depletion of chitin in *T. castaneum* [7]. Injection of the *UAP* gene 20E regulated *S. exigua* and raised the *SeUAP* transcript [56]. Taken together, further research needs to determine the impacts of dsRNA on these genes and observe the interactions between arthropod reproduction, JH hormones, and various enzymes of the biosynthetic chitin pathway.

To obtain further understanding of the functions of the *chitin synthase 1* gene regarding maintaining chitin, transmission electron microscope (TEM) analysis was performed on pharate nymph and pharate adults, and a lower laminar organization was observed after RNAi of *CHS1*. Similar results were also observed in a specific region of the body wall, which is associated with a denticle-like structure. These denticles are interlocked with a subsequent layer of denticles from the inner-side. The Velcro-like structure has a hooked structure, which has a significantly important role in a specific region of the trachea in interactions with similar fibers and firm snapping. In addition, an electron-dense material accumulates under these folds. Our findings can also be justified by previous reported research, wherein denticles were shown to be completely degraded after treatment with chitinase dsRNA, compared to a control group [57].

## 5. Conclusions

The present study revealed that the *CHS1* gene, when suppressed with RNAi, inhibits chitin content, which ultimately leads to mortality in the target pest. Disturbance in chitin also had an effect on the female fertility of the *P. citri*. Our results help to illuminate the molecular mechanism and function of the *CHS1* gene, which impacts fecundity in adult *P. citri*. Using the leaf dip method, our results reveal that dsRNA was effective and significantly reduced the egg-laying potential and hatching rate of *P. citri* eggs. These findings indicate the potential utility of the *CHS1* gene in the development of novel RNAi strategies for controlling *P. citri*. The future research should focus on the optimization and application in order to develop economically feasible and effective citrus red mite control measures with using molecular acaricide.
